# Supplementary material for: Remote ischemic preconditioning does not influence lectin pathway protein levels in head and neck cancer patients undergoing surgery
Source: PLoS One. 2020 Apr 8;15(4):e0230411. doi: 10.1371/journal.pone.0230411 (PMC7141620; doi:10.1371/journal.pone.0230411)
Supplement: Attachment — Submitted filename: Response to Reviewers.docx [file pone.0230411.s004.docx]

We thank the editor and reviewers for the very relevant comments, which have supported improvement of the manuscript. Below, please find our point-by-point response.

Journal Requirements:

When submitting your revision, we need you to address these additional requirements.

**Comment 1.** Please ensure that your manuscript meets PLOS ONE's style requirements, including those for file naming. The PLOS ONE style templates can be found at

http://www.journals.plos.org/plosone/s/file?id=wjVg/PLOSOne_formatting_sample_main_body.pdf and http://www.journals.plos.org/plosone/s/file?id=ba62/PLOSOne_formatting_sample_title_authors_affiliations.pdf

**Answer 1:** We have made sure that the manuscript meets PLoS ONE’S style requirements, including the requirements for file naming.

**Comment 2.** Thank you for submitting your clinical trial to PLOS ONE and for providing the name of the registry and the registration number. The information in the registry entry suggests that your trial was registered after patient recruitment began. PLOS ONE strongly encourages authors to register all trials before recruiting the first participant in a study.

As per the journal’s editorial policy, please include in the Methods section of your paper:
i) your reasons for your delay in registering this study (after enrolment of participants started);
ii) confirmation that all related trials are registered by stating: “The authors confirm that all ongoing and related trials for this drug/intervention are registered”.

Please also ensure you report the date at which the ethics committee approved the study as well as the complete date range for patient recruitment and follow-up in the Methods section of your manuscript.

**Answer 2:** We thank the editor for pointing out this issue regarding mismatch between trial registration date and the stated inclusion period in the manuscript.

The trial was registered on ClinicalTrials.gov on September 14, 2015. As requested, we have added the following sentence in the Methods section: “The authors confirm that all ongoing and related trials for this intervention are registered.” (P7, L123-124).

We realized that we have made an error in the Results section stating that patients were included between August 2015 and November 2017. The inclusion period was in fact September 29, 2015 to November 28, 2017, which has been corrected in the revised manuscript (P11, L234-236).

The trial was approved by the Central Denmark Region’s Ethics Committee on August 13, 2015. We have added this date to the Materials and methods section (P7, L120-121).

Further, we have specified the inclusion period and follow-up period in the beginning of the Results section in the following sentence:

“Sixty patients were enrolled between September 29, 2015 and November 28, 2017; 30 patients in the RIPC group and 30 in the sham group, as described in [32]. 30-day follow-up was completed on December 28, 2017” (P11L234-236).

**Comment 3.** We noticed you have some minor occurrence(s) of overlapping text with the following previous publication(s), which needs to be addressed:

https://doi.org/10.1371/journal.pone.0219496
https://doi.org/10.1111/aji.13092
https://doi.org/10.1111/sji.12680

In your revision ensure you cite all your sources (including your own works), and quote or rephrase any duplicated text outside the Methods section. Further consideration is dependent on these concerns being addressed.

**Answer 3:** Thank you for making the iThenticate report available for us. We were a bit puzzled about this issue. We checked our previous manuscript and found only very little overlap and we have sited all previous work thoroughly. However, we realized some duplicate texting with our previous conference abstract accepted at the International Society of Thrombosis and Hemostasis. Subsequently, we have rephrased the abstract, in the manuscript which contained the major part of the duplicate texting.

Further we have made minor rephrases in the Methods section, Results and Discussion.

[Note: HTML markup is below. Please do not edit.]

Reviewers' comments:

Reviewer's Responses to Questions

**Comments to the Author**

1. Is the manuscript technically sound, and do the data support the conclusions?

The manuscript must describe a technically sound piece of scientific research with data that supports the conclusions. Experiments must have been conducted rigorously, with appropriate controls, replication, and sample sizes. The conclusions must be drawn appropriately based on the data presented. 

Reviewer #1: Yes

Reviewer #2: Yes

2. Has the statistical analysis been performed appropriately and rigorously? 

Reviewer #1: N/A

Reviewer #2: Yes

3. Have the authors made all data underlying the findings in their manuscript fully available?

The [PLOS Data policy](http://www.plosone.org/static/policies.action#sharing) requires authors to make all data underlying the findings described in their manuscript fully available without restriction, with rare exception (please refer to the Data Availability Statement in the manuscript PDF file). The data should be provided as part of the manuscript or its supporting information, or deposited to a public repository. For example, in addition to summary statistics, the data points behind means, medians and variance measures should be available. If there are restrictions on publicly sharing data—e.g. participant privacy or use of data from a third party—those must be specified.

Reviewer #1: Yes

Reviewer #2: Yes

4. Is the manuscript presented in an intelligible fashion and written in standard English?

PLOS ONE does not copyedit accepted manuscripts, so the language in submitted articles must be clear, correct, and unambiguous. Any typographical or grammatical errors should be corrected at revision, so please note any specific errors here.

Reviewer #1: Yes

Reviewer #2: Yes

5. Review Comments to the Author

Please use the space provided to explain your answers to the questions above. You may also include additional comments for the author, including concerns about dual publication, research ethics, or publication ethics. (Please upload your review as an attachment if it exceeds 20,000 characters)

Reviewer #1:

**Comment 1.** I appreciate the opportunity to review your revised manuscript. The topic is of Novelty and of clinical importance. (P refers to page, L referes to line, for examole,P10L191-196 refers to Page 10 line 191-196）.

P10L191-196, “The outcomes were: 1) ……”. In clinical trial, there are primary outcome and with or without secondly outcomes, the primary outcome is usually the most interested outcome and relative to sample calculation. Therefore, I would suggest pointing out which outcome was primary outcome.

**Answer 1:** We agree with the reviewer that the primary outcome should be specified. We have rewritten the Outcomes section, so it now reads:

P10L197-203:

“The primary outcome was: difference in MASP-2 protein concentration between the RIPC and sham group on the first postoperative day.

The secondary outcomes were: 1) difference in LP protein plasma concentrations between healthy individuals and head and neck cancer patients before surgery; 2) difference in LP protein concentration changes between the RIPC and sham group from before surgery to after surgery; and 3) difference in postoperative LP protein concentration between patients who developed surgical site infection and patients who did not.”

In accordance with this, we have included the following analysis in the Results section:

P13L275-277:

“There was no difference in MASP-2 concentrations on the first postoperative day between the RIPC and sham group (RIPC: mean 461 (SD: 212) ng/ml vs. sham: mean 490 (SD: 140) ng/ml, (*P* = 0.53)).”

**Comment 2.** P10L199-200, “A sample size calculation was conducted for the study on hemostasis[32], which defined the number of participants in the present study ”. It seems that the sample size was calculated the same as the article mentioned. But I read the article mentioned , and find the sentence，“The sample size was calculated with collagen-induced platelet aggregation (COL test，Roche Diagnostics) on the first postoperative day as primary endpoint”，since the outcomes were totally not the same in the two trial, I don’t think the sample size calculation could be the same. So, to make it clere that how sample size was calculated would be more appropriate.

**Answer 2:** We appreciate the point made by the reviewer and apologize that we missed to include the sample size calculation for the present study in the first version on the manuscript. We have added the following information from our pre-specified protocol in the section on sample size and statistical analysis:

P10-11L206-213:

“Plasma levels of lectin pathway proteins in head and neck cancer patients have not been

described previously. However, data from a randomized controlled trial

including lung cancer patients showed a mean postoperative MASP-2 plasma level of 440 ng/ml with a standard deviation of 133 ng/ml [41]. We estimated the minimal relevant difference to be 120 ng/ml. With an α (two-sided) of 0.05 and power (1 – β) of 90%, we would be able to detect a statistically significant difference in MASP-2 postoperatively, if 26 patients were included in each group. We included 30 patients in each group to account for potential drop-outs or missing data.”

Reviewer #2:

**Comment 1.** Interesting paper dealing with "Remote ischemic preconditioning" and lectin pathway protein levels in head and neck cancer patients undergoing surgery.
While it is a good idea to use ischemic preconditioning in these patients no effects were observed.
Might be due to heterogeneous patients or small patient number though. The authors should emphasize this a bit more in the introduction and discussion section.

**Answer 1:** We sincerely thank the reviewer for the appreciative comment. We agree with the reviewer that this it be emphasized more that the negative finding of no effect could partly bed due to heterogeneous patients or small patient number. In accordance, we have added a sentence in the limitation section of the Discussion, which reads:

P19-20L417-419:

“Likewise, we cannot rule out that minor differences between groups have been missed due to the relatively small sample size or heterogenieity in the included patient group.”

6. PLOS authors have the option to publish the peer review history of their article ([what does this mean?](https://journals.plos.org/plosone/s/editorial-and-peer-review-process#loc-peer-review-history)). If published, this will include your full peer review and any attached files.


If you choose “no”, your identity will remain anonymous but your review may still be made public.


**Do you want your identity to be public for this peer review?** For information about this choice, including consent withdrawal, please see our [Privacy Policy](https://www.plos.org/privacy-policy).

Reviewer #1: No

Reviewer #2: No


[NOTE: If reviewer comments were submitted as an attachment file, they will be attached to this email and accessible via the submission site. Please log into your account, locate the manuscript record, and check for the action link "View Attachments". If this link does not appear, there are no attachment files to be viewed.]


While revising your submission, please upload your figure files to the Preflight Analysis and Conversion Engine (PACE) digital diagnostic tool, <https://pacev2.apexcovantage.com/>. PACE helps ensure that figures meet PLOS requirements. To use PACE, you must first register as a user. Registration is free. Then, login and navigate to the UPLOAD tab, where you will find detailed instructions on how to use the tool. If you encounter any issues or have any questions when using PACE, please email us at[figures@plos.org](mailto:figures@plos.org). Please note that Supporting Information files do not need this step.
